# Supplementary material for: Engaging community members in setting priorities for nutrition interventions in rural northern Ghana
Source: PLOS Glob Public Health. 2022 Sep 16;2(9):e0000447. doi: 10.1371/journal.pgph.0000447 (PMC10022374; doi:10.1371/journal.pgph.0000447)
Supplement: S1 Table — (DOCX) [file pgph.0000447.s001.docx]

**S1 Table : Policy documents reviewed**

| **Policy** | **Start** | **End** | **Adoption** | **First 1000 years of life** |
| --- | --- | --- | --- | --- |
| Under-five’s child health policy | 2007 | 2015 | Adopted | Yes |
| Breastfeeding Promotion Regulations 2000, LI1667 | 2000 |  | Adopted | Yes |
| Child health policy | 2007 | 2015 | Not Adopted | Yes |
| Breastfeeding Promotion Regulations | 1995 |  | Adopted | Yes |
| Infant and Young Child Feeding Strategy for Ghana | 2007 |  |  | Yes |
| Vitamin A Policy | 1998 |  | Adopted | Yes |
| National Nutrition Policy | 2014 | 2017 | No | Yes |
| National Nutrition policy | 2016 |  |  |  |
| National health Policy | 2007 | 2011 | Adopted |  |
| VIT A policy nutrition policy or strategy | 1998 |  | adopted |  |
| Integrated Nutrition Policy | 2003 |  | adopted |  |
| National Reproductive Health Service Policy and Standards | 2003 |  | adopted |  |
| National Plan of Action on Food and Nutrition | 1995 | 2000 | adopted |  |
