# Supplementary material for: Engaging community members in setting priorities for nutrition interventions in rural northern Ghana
Source: PLOS Glob Public Health. 2022 Sep 16;2(9):e0000447. doi: 10.1371/journal.pgph.0000447 (PMC10022374; doi:10.1371/journal.pgph.0000447)
Supplement: S1 Text — (DOCX) [file pgph.0000447.s003.docx]

**S1 Text : Facilitator Script**

## **CHAT**

### “**C**hoosing **A**ll **T**ogether”- for **N**utrition

*Copyright © 2005. The Board of Regents of the University of Michigan.*

*All Rights Reserved*

*This version of CHAT was prepared by INPREP Group in collaboration with NIH funded by the National Institute for Health Research (NIHR) (17\63\154) using UK aid from the UK Government to support global health research.*

Note to Facilitator: Read the regular black print out loud. The blue italicized print in parentheses provides the facilitator directions that are not meant to be read aloud.

**Introduction**

*(With the help of the fieldworkers, prepare the room as follows: Place chairs around a table in a circle. Place the large CHAT board in the middle of the table (or in sight of all participants) and have 60 stickers ready to use on the table. Place 2 voice recorders at 2 ends of the table, have the recorder ready to be turned on. Place a participant information sheet, consent form at each seat, and a small CHAT board at every second seat.)*

**STUDY TITLE:** Making decisions for better nutrition

Welcome! I’m _______________ and these are my research assistants, _________________. Today you are taking part in an exercise called “CHAT.” CHAT stands for CHoosing All Together. We are here to help you decide what nutrition programmes are most important to your community.

You have each received a participant information sheet from the research assistants when they invited you to participate in the study. You have signed a consent form to participate as well as a form that consents to the audio recording of the exercise today. I would like to remind you that you will not be expected to share your name and it will not be connected with the recording or the transcript. You will not be identified by name in any of the reports or publications of this study or its results. The information that is collected will be used for educational and scientific purposes.

Taking part in this study is completely your choice. You have already agreed to participate but you can stop participating in the study at any time if you want. By agreeing to participate you must feel comfortable with the exercise being audio-recorded and if you are not comfortable with this you are free to decline/withdraw before or even after you have started the process. If you say no, this will not have any negative effect on you in any way whatsoever.

This exercise is the second step of a bigger research project. In the first step we asked community members about issues and solutions linked to nutrition. This helped us identify and develop a number of nutrition programmes. Today we will try to understand which of these nutrition programmes are most important to you as a group.

This project is an opportunity for you to voice your views about nutrition. Thank you for taking the time to share this experience with us. We will answer all your questions as we go along. We hope you have fun.

You have each been given a sticker with a participant number. Please stick this on your shirt, we will use this to refer to you during the exercise. This way we will protect your privacy. We ask all of you to also keep private information you hear from each other during the game –private; please do not repeat what you heard after you leave this session.

OK, now as we begin the CHAT exercise, let me tell you a bit about the nutrition programmes that we are going to discuss, and you will make decisions about.

Sometimes mothers and children in your household are malnourished and become ill. When this happens, you try to help them as best you can. You try to feed them more food or take them to the clinic to get medicine. If you do not have access to enough food, or you cannot go to the clinic and get medicine, your family member may be ill longer. If the illness gets terribly bad, your family member must go to the hospital. Some children will suffer irreversible health damage such as blindness due to malnutrition. The government tries to provide nutrition programmes to make sure that mothers and children are able to get the nutrition care and support that they need. The government also tries to stop mothers and children becoming malnourished in the first place by running things like education campaigns. All these things cost the government money and, just like in every country in the world, there sometimes isn’t enough money to pay for all the services that we need. The government needs to decide which health services are the most important and where they should spend their money. It’s the same as when you go to the shops. You have some money, and you go to the markets to buy food for your family. You don’t have enough money to buy everything, so you need to decide between pawpaw, cabbage, milk, meat etc. These are the choices that you make. In the same way, the government has to choose which nutrition programmes to provide.

So, in this exercise we want you to think about which nutrition programmes you would like to see included in a nutrition package. For example, it could be nutrition education through a media campaign, or community groups with demonstrations of healthy and affordable recipes using locally available ingredients. You may think things like agricultural support, or food fortification is important. You will make choices using the stickers that are available to you. The stickers are the money that is available, and you will only be able to select as many options as the stickers can cover. We hope this exercise will help you to understand that not everything can be provided and will help you to choose the things that are most important. There are no right or wrong answers.

How does everyone feel about this so far? What questions do you have?

As we begin, let me give you some instructions:

**Purpose**

The purpose of this CHAT exercise is to help the group decide together which nutrition programmes are the most important.

#### Taking part in CHAT

There are four parts in this CHAT exercise.

- The first part is a practice round. In this **first CHAT Round** you will work in pairs, and you will make a nutrition package that you think is best for your community.
- In the second part each of you will receive a **nutrition scenario card** that will explain some of the consequences of the nutrition programmes.
- The third part is the **second CHAT Round**. You will all work together to make a nutrition package for a whole community.
- The fourth part is to discuss with you how the CHAT exercise went and how you found working together about nutrition in your community.

Before we begin, let’s start with some ground rules to set so we can make the most of this exercise. How do you think we should all interact with each other in this session? *(This part is to get the community members to make their own rules, examples include treat everyone with respect during this exercise, give other people a chance to talk, please do not interrupt others or have your own conversations on the side).*

*OPTION: Before we start the CHAT activity, we would briefly like to discuss what you think are the nutrition issues in the community. This will help us in later discussions about the nutrition programmes.*

*(Possible questions to ask*

- *What do you think are the reasons for nutrition issues in your community?*
- *What are the main barriers to eating healthy food?*
- *What past programmes worked well for your community?)*

**FIRST PART – In pairs you determine your ideal Nutrition Package for your community**

Let’s start with the practice round. Now let’s turn to our neighbour on the right side. Is there anyone who does not have a partner? *(Allocate a research assistant to anyone without a partner)* We are giving each pair a small version of the CHAT Board and stickers. *(Research assistants to distribute one A4 CHAT Board per pair).*

Does everyone have a **CHAT Manual?** *(Hold up* ***CHAT Manual****.)* The **CHAT Board** has **6** areas, all linked to nutrition. Some of these areas have different nutrition porgrammes to choose from, for example- within the **community nutrition** area there is radio broadcasting or food demonstrations. I will show you the picture, the color, and the name of each of the areas *(Hold up the CHAT Board and point to icon and name of each service area while reading the names out loud.) (Hold up* ***CHAT Manual****.)* Your **CHAT Manual** explains these different areas and programmes. *(Open the CHAT manual, point to the icon for each area and read the description of each option).*

Before you decide which programmes to pick, the research assistants will read the CHAT board manual which explains the different components of the programmes. During the game if anyone needs help looking for things in the manual please ask and we will help.

*(Hold up* ***Stickers****.)*  You make your choices by pasting stickers on the CHAT Board. This is why we give you **60 Stickers** to paste. You can only choose nutrition programmes so long as you have stickers, so the game is to choose the programmes you want most with the number of stickers you have.

For each nutrition programme you might choose to take it or leave it. Some programmes do not cost very much and only need 1 sticker while others cost more and need more stickers (Demonstrate the different options on the CHAT Board.).

*(Possible questions to ask the group to check their understanding*

- *What is the aim of this activity?*
- *How will you identify nutrition programmes to tackle community issues?*
- *How will you work together to identify nutrition programmes for your community?)*

Let’s begin the **practice round**. You will each take turns saying what you prefer, and try to convince your partner to agree. You have to tell your partner why you make this choice, and allow them to comment. You will decide together whether it is a good idea to add the option and whether to include it. Once you agree, you will place stickers on the CHAT Board. You and your partner can decide to change your mind and move around your stickers at any point. You will work on your own in pairs but the research assistants will move around the room to provide support in using the board and the manual.

How is everyone feeling about the exercise? What questions does anyone have?

You can take about 25 minutes for this step. Go ahead; begin.

(PAUSE. Allow pairs time to work. Circulate to monitor how the pairs are doing and identify those who are having difficulties. Be available to answer questions. Let players know when there are only a few minutes left. After about 25 minutes say)

**SECOND PART - Test Your Nutrition Package with Nutrition Scenario Cards**

Okay. Now that you finished making your choices you can’t change them anymore in this round. Let’s test your package. You can see the results of the choices you picked by the CHAT scenario cards which the research assistants will read. Each of the cards relates to one of the nutrition programmes on the board. On the back of the card there is a nutrition problem you might face. The research assistants will read the cards. Some Scenario Cards are about men or women or children.

After we read the scenario card, please share your thoughts about how the nutrition package you and your partner have designed in the practice round would help the nutrition problem or not and would you change anything about your package. As you listen to the different nutrition stories on each card, you’ll get a better and better idea of what is important and what you want the nutrition package to include.

***Now the research assistants will read the cards*** *(and facilitate a discussion around the questions using the probe below)*

(***PAUSE.*** *Probe with additional questions such as:* *What did you and your partner choose? What impact would those programmes have on the scenario card? How much did your package work the way you expected it to? After hearing the nutrition scenario cards, how likely are you to change your mind and what would you change your mind to? After completion of Second Part, go to Part Three)*

##### PART THREE - Work together as a group to choose your ideal nutrition programmes for your whole community

We’ve completed the practice round and will now move on to the Group Round. Now we’ll “Choose All Together” a nutrition package that is for everyone in the community. To do this, we’ll use this Big CHAT Board *(Point to Board).*

We’ll each take turns saying what programmes we prefer and try to work together to come to a consensus. We’ll go around the table asking each person to choose a nutrition programme within one of the areas. We will ask you to tell us what made you choose this programme and allow others to comment. If someone disagrees with a suggestion, please raise your hand and tell us what you think. You will decide together whether it is a good idea to add the option and you will vote whether to include it. If it gets a majority vote – over 50% -, I’ll place stickers on the CHAT Board. Once we used up all the stickers, there is still a chance to make changes by moving stickers around. Please don’t be upset if what you want does not get included in the board- remember the way we decide is by majority vote.

*Any questions before we start? (PAUSE* *for questions*.)

Let’s begin making our recommendations. *(Pick a person to start.)* Let’s begin with you. What nutrition area and which programme would you like to select? What made you select this? *(important to remember to ask what the reasons are for the participants selecting the programmes that they do and for research assistants to note. Possible questions to ask each individual:*

- *What made you choose this programme?*
- *How comes this programme is important to you?*
- *How would this programme impact your or your family’s lives?)*

*(Once a participant says the programme that s/he would prioritise, you can ask other people what they think. Possible questions include:*

- *What do you think of his/her programme choice?*
- *What do other people think?*
- *How many people agree/disagree with PARTICIPANT NUMBER’s choice of programme?*

*If it gets a majority vote – over 50% -, you can place stickers on the CHAT Board. Each time, say how many stickers are left.)*

*(Once everyone has had a turn and the stickers are used ask the group if they are satisfied with the group choices on the board, if anyone wants to suggest a modification to the choices they can but must provide justification and must be approved by majority vote. Possible questions to ask the group:*

- *How satisfied are you with the choices on the board?*
- *Who would like to make any modifications, if so what changes?*
- *What do other people think about the proposed modification?)*

*What nutrition programmes have you decided on as a group?*

*Summarize the discussion and read out the final programmes of choice on the CHAT Board.*

Thank you for participating in the CHAT exercise. We hope you enjoyed it. Before we leave to go, we have a few questions we would like to ask you in PART FOUR.

**PART FOUR – Short-term impact of working together about nutrition in their community**

- How do you feel about participating in this exercise?
- How much do you feel you have contributed to the discussions today?
- How did you find the activity (what went well/what didn’t go well?
- What did you think of working together as a group today?
- How do you think you could work together to improve nutrition for your community?
- What’s your next step to work together to improve nutrition for your community?

Thank you for your important insights about the nutrition programmes that you would prioritise in your community. We hope to discuss your priorities with your local stakeholders in the future. Thank you for your time.

*Possible facilitator questions that you might want to ask the group:*

- *How hard or easy is the decision to select these programmes?*
- *What are the possible consequences of this programme on families and communities?*
- *What would happen if women spent more time supporting the men with agriculture programmes?*
- *What are the possible consequences of this programme on your community setting?*
- *How would that programme work in your community?*
- *What parts of the programme would work?*
- *What parts of the programme wouldn’t work?*
