# Supplementary material for: Engaging community members in setting priorities for nutrition interventions in rural northern Ghana
Source: PLOS Glob Public Health. 2022 Sep 16;2(9):e0000447. doi: 10.1371/journal.pgph.0000447 (PMC10022374; doi:10.1371/journal.pgph.0000447)
Supplement: S2 Text — (DOCX) [file pgph.0000447.s004.docx]

**S2 Text: Nutrition scenario cards**

**LIVELIHOOD EMPLOYMENT**

| A friend, Akulpoka lives with her two children. She has been struggling to provide for her family and her children are hungry. This week she had to sell assets to buy food. If Akulpoka picked livelihood skills training, she would be able to learn how to produce and sell her own vegetables. She could prepare nutritious meals for her children. |
| --- |

| Adua, a young father lives with her wife and a 15-month-old daughter, Kawe. Adua keeps chickens but cannot grow a vegetable garden because rain has been erratic, and he does not have running water. For the past few months, Adua’s wife has been feeding her daughter a thin porridge, but in the last two weeks Kawe has not been eating well. She was admitted to the hospital and was diagnosed with severe wasting, and dehydration. If you picked water wells and water tanks Kawe’s parents would have access to water all year round and could produce and prepare nutritious meals. |
| --- |

**MICRONUTRITENT SUPPMENETATION**

| Nsorma, an 18-month-old girl, lives with her grandmother an hour from the Hospital by bus. Nsorma was breastfed for one week, but thereafter her mother returned to work as a domestic worker in the city. For the past few months, Nsorma’s grandmother has been feeding her a thin porridge, but in the last two weeks she has not been eating well. She was admitted to the hospital and was diagnosed with severe wasting, and dehydration. If you picked the home fortification, Nasorma’s grandmother would have been given supplements and Nsorma would have been given the right nutrients. |
| --- |

| A friend, Wepia has given birth to low-birth weight baby. Before pregnancy, Wepia has been feeling very tired, she had trouble with her attention, memory and her work performance dropped. At the hospital, the nurse said she has not been getting the right food and is low in iron. She tested her iron level and she was anemic. If you picked iron supplementation Wepia would have gotten supplements from the clinic and advice from the nurse on what to eat would have given birth to a healthy child. |
| --- |

**NUTRITION EDUCATION**

| Awenlie has given birth to a child with disability. During her pregnancy, Awenlie was drinking alcohol and missed most of her ANC visits. At the hospital she was told that drinking alcohol put her baby into risk of birth defects. If you picked nutrition education through SMS, Awenlie would have received reminders of the importance of ANC visits and messages about the harms of alcohol at any time during pregnancy on her baby’s development. Awenlie would have given birth to a healthy child. |
| --- |

**HEALTH SYSTEM STRENGTHENING**

| A friend’s child, Wedam has been feeling very tired over the last few months. He has been getting in trouble at school for not listening in class. Wedam has also been saying his eyes are sore and that he can’t see too well. Wedam’s mum did not have enough the resources at the time and delayed the clinic visit. When his mother took him to the clinic the nurse said he has not been getting the right food and is low in iron and vitamin A. They tested his eyes and he is blind in one eye and can only see a little bit in the other eye. If you picked the training of CHV, Wedam would have gotten advice from the CHV at home on what to eat would have saved Wedam from being blind. |
| --- |

**MEN INVOLVEMENT**

| One day your neighbour Mmalebna came to your house to tell you that she found her 3-year-old daughter weak, pale and food deprived. The child was left alone at home with Mmalebna’s husband. She thinks the father may have neglected the daughter and not fed her appropriately. If you picked the male involvement in nutrition education there would be community groups organized to help men learn about how to take care of pregnant women and children. Engaging fathers effectively in nutrition may have helped Mmalebna’s husband to feed the child appropriately. |
| --- |
