# Supplementary material for: Engaging community members in setting priorities for nutrition interventions in rural northern Ghana
Source: PLOS Glob Public Health. 2022 Sep 16;2(9):e0000447. doi: 10.1371/journal.pgph.0000447 (PMC10022374; doi:10.1371/journal.pgph.0000447)
Supplement: Attachment — Submitted filename: Response to Reviewers.docx [file pgph.0000447.s005.docx]

University of Health and Allied Sciences

Institute of Health Research

PMB 31

Volta Region

Ho

Ghana

1^st^ February 2022.

Dear editor,

We appreciate the opportunity to resubmit our manuscript entitled “Engaging community members in priority setting for nutrition interventions in rural northern Ghana” for publication in Plos Global Public Health. We would like to thank the editors and reviewers for the useful comments. We have addressed all comments raised, with corresponding changes made to the manuscript where appropriate. Detailed responses to the comments are presented below:

**Addressed editors comments**

Journal Requirements:

1. Please provide separate figure files in .tif or .eps format only, and remove any figures embedded in your manuscript file.  If you are using LaTeX, you do not need to remove embedded figures.

For more information about figure files please see our guidelines: <https://journals.plos.org/globalpublichealth/s/figures>

*Response*

*Figures are not embedded in the manuscript and are presented separately in the appropriate file format*

2. Please amend your detailed Financial Disclosure statement. This is published with the article, therefore should be completed in full sentences and contain the exact wording you wish to be published.

i) Please include all sources of funding (financial or material support) for your study. List the grants (with grant number) or organizations (with url) that supported your study, including funding received from your institution. 
ii). State the initials, alongside each funding source, of each author to receive each grant.
iii). State what role the funders took in the study. If the funders had no role in your study, please state: “The funders had no role in study design, data collection and analysis, decision to publish, or preparation of the manuscript.”

*Response: We have now amended the financial disclosure in the article submission.*

**Reviewers' comments:**

**Reviewer 1**

General comments

Above mentioned descriptive cross-sectional study perfectly describes that priority setting for ‘nutrition interventions’ is possible and feasible by engaging community members with the aid of a simulation decision-making tool namely ‘Choosing All Together’ (CHAT) in a rural setting of low literacy and resources. The manuscript is well organized, so is the methodology which can be adopted in resource-poor setting with higher malnutrition prevalence, where prioritizing is important for funding in the area of nutrition health policy strategy and implementation. However, some minor unintended errors (especially numbering references in the text) are observed in some places of the manuscript. I strongly recommend to accept this paper with some minor corrections.

Specific comments

Introduction

Between line 100 and 101: Instead of giving reference number 3 (Tugendhaft et al. 2021) authors (with colleagues) names are written; however, together with Tugendhaft et al. 2021, name of Hurst et al. 2018 (wrongly?) is also mentioned which has reference number 7 in reference list, but serially next references are 4, 5 (in the line 104), and then references 6, 7 are in the line 111 (along with reference 3).

Authors carefully should resolve above mentioned problems along with reference list.

*Response*

*References have been corrected*

Material and Methods

Study area

Between line 130 and 131: Instead of giving reference number 10 (Dalaba et al. 2016) and 11 (Oduro et al. 2012), authors (with colleagues) names are written without closing round bracket like ---Dalaba etal. 2016; Oduro et al. 2012).

*Response*

*The references have been corrected*

Study population and sampling

Line 153: Should elaborate first e.g. In depth interviews (IDIs), though it is elaborated and mentioned later in the line 195.

*Response*

*FGD and IDI has been written in full. The sentence now reads:*

*For the identification of relevant policies and interventions, study participants constituted community members and stakeholders in health, nutrition and maternal and child health as well as non-governmental organizations. Stakeholders were identified and In-Depth Interviews (IDIs) carried out with them. Community members sampled from the study communities were scheduled and grouped for Focus Group Discussions (FGDs).*

*(location of revision: Page 7, line,388-392)*

Results

Socio-demographic characteristics of participants

Line 305: table 1 should also be mentioned in the parenthesis as table 2 has no age related data

*Response*

*Age group has been added in Table 2*

Discussion

Line 497: after the line ‘constructed to boost agricultural activities when voted into power’ insertion of number (30) (reference?) does not make any sense as reference 30 does not exist in reference list.

*Response*

*The reference has been added*

**Reviewer #2:**

In this study, which aims to identify priorities in nutrition interventions and the reasons for choices in communities living in Northern Ghana, what makes the study important is that it supports the importance of community participation in priority decisions to improve services in communities.
Below you can find some arrangements for organizing the writing of the work specifically to improve the materials and methods, results and discussion.

INTRODUCTION
The citations on lines 100-101 should be corrected and reviewed in the references accordingly.
The introduction should be expanded by briefly mentioning similar practices in other countries.

*Response*

*References have been corrected and also details other previous studies in other countries have been added.*

*“For instance, CHAT was implemented in a rural community in South Africa to determine priorities for a health services package. The study demonstrated that deliberative engagement methods can be successful in helping communities balance trade-offs and in eliciting social values around health priorities(3). Similarly, CHAT was conducted among residents of Switzerland to discuss priorities for Swiss Health insurance coverage. Results showed that participants were able to engage with complex resource allocation trade-offs in healthcare coverage and to agree on a set of priorities(4)”*

*(location of revision: Page 5, line,330-336)*

MATERIAL AND METHOD
The citations on lines 130-131 should be corrected and should be reviewed in the same way.
It should be defined where the abbreviations are first mentioned and should be used optionally as abbreviations in the following sections. (such as FGD and IDI abbreviations)
Other abbreviations should be checked.

*Response*

*References have been corrected and FGD and IDI have been written in full. The sentence now reads:*

*For the identification of relevant policies and interventions, study participants constituted community members and stakeholders in health, nutrition and maternal and child health as well as non-governmental organizations. Stakeholders were identified and In-Depth Interviews (IDIs) carried out with them. Community members sampled from the study communities were scheduled and grouped for Focus Group Discussions (FGDs).*

*(location of revision: Page 7, line,388-392)*

DISCUSSION
484-485 lines. This section should be expanded by scanning the literature for different age groups in the lines.

Response

This has been expanded and now reads:

*“The seasonal effect of food insecurity leads to higher risk of severe acute malnutrition among children aged less than three years old (21–23). For instance, a previous study conducted in the study area revealed that being born in the hunger season is associated with a higher risk of severe acute malnutrition (23). Therefore there is the need for improvement in the food supply to pregnant and lactating mothers through livelihood empowerment interventions and sustainable agriculture in order to reduce the burden of severe acute malnutrition”*

*(location of revision: Page 23-24, line 938-966)*

488-490 lines should be referenced.

Response

Reference has been added

500-506 lines should be expanded with studies on this subject, especially based on the current literature.

Response

This has been expanded to include a study on nutrition. This reads as:

*“*

*“A previous study showed that long-term routine use of micronutrient powder containing prophylactic iron reduced anaemia, iron deficiency and iron deficiency anaemia among pre-school children living in rural Ghana (27). A recent Lancet review confirmed that micronutrient supplementation was an effective intervention to improve maternal and child nutrition(28)”*

*(location of revision: Page 24, line,985-989)*

507-517 lines studies on male participation should be added and expanded.

A study has been added and it reads;

*“In a study conducted in Ghana on male involvement in maternal healthcare, it was concluded that sustenance of male involvement especially, husbands is required at the household and community levels for positive maternal outcomes(31)”.*

*(location of revision: Page 25, line,1066-1068)*

Limitations of the study should be added.
It is recommended to mention the strengths of the study and suggestions for future research in the conclusion.

Response

The strengths and Limitations of the study has been added

***“Strengths and limitations of the study***

*One important strength of this study is that the process of identifying the prevailing nutrition interventions was robust. It included the triangulation of evidence from lay and published literature, interaction with different stakeholders and the involvement of the community.*

*The second strength is the use of lay community members to pilot CHAT and prioritise nutrition interventions in the face of diverse intervention options with limited resources. This gave the opportunity for community members to appreciate the processes involved in prioritising and implementing health interventions. The implementation process also gave insights to the community members of public deliberation exercise and overall the insights on setting priorities in intervention. It might also lead to a sense of appreciation among community members and improved adherence to existing interventions haven appreciated the associated implementation cost. The successful adoption, modification and pilot implementation of CHAT for nutrition interventions and in a rural setting is a major strength to our study. It implies that, the methodology used can be scaled up to engage more groups and replicated in other settings in Ghana. The tool perhaps may be modified and used to prioritise other health related outcomes beyond nutrition. This premier study has generated reliable results and may serve as a benchmark for future CHAT related studies in Ghana and elsewhere in sub-Saharan Africa.*

*Despite these strengths, we would like to acknowledge a few limitations to our study. First, the lengthy time commitment from participants to undertake the CHAT exercises. This was a considerable inconvenience to some participants since it took an average of 3 hours. Given that the participants could not read the instructions, a lot of time was spent by the facilitators in explaining the exercise to participants in the local language. Nevertheless, participants appeared to find the CHAT sessions interactive and intriguing as facilitators were able to maintain the focus of the participants throughout the exercise.*

*CHAT instructions were translated from English into the local language. It is possible that the intended meaning of some of the instructions may have been lost during the translation process. However, the facilitators were experienced research officers who are natives to the area, thus minimizing the potential for mistranslations making this unlikely to affect the study findings.*

*Another limitation of the study is the small number of groups (6 groups) used for the pilot implementation of the CHAT exercise which we believe may not be a representative of the studied population. This may limit the generalizability of our findings to the study population”*

*(location of revision: Page 26-27, line,1148-1184)*

REFERENCES
It should be reviewed by paying attention to the abbreviations in the journal names (references such as 13, 14, 15)
There appear to be two 29th sources, should be checked.

*Response*

*References have been updated*

The authors will be happy to clarify further issues that the reviewers may raise. The manuscript has been read again and all grammatical errors has been corrected to improve the quality of the manuscript. We hope that the concerns raised are adequately addressed.

Accompanying this response letter is a revised version of our manuscript.

**Sincerely,**

Maxwell Dalaba (on behalf of the authors)
